# Refining animal models in fracture research: seeking consensus in optimising both animal welfare and scientific validity for appropriate biomedical use

**DOI:** 10.1186/1471-2474-8-72

**Published:** 2007-08-01

**Authors:** Jorg A Auer, Allen Goodship, Steven Arnoczky, Simon Pearce, Jill Price, Lutz Claes, Brigitte von Rechenberg, Margarethe Hofmann-Amtenbrinck, Erich Schneider, R Müller-Terpitz, F Thiele, Klaus-Peter Rippe, David W Grainger

**Affiliations:** 1 Equine Hospital, Vetsuisse Faculty University of Zurich, Winterthurerstrasse 260, CH-8057 Zurich, Switzerland; 2 Royal Veterinary College and Institute of Orthopaedics and Musculoskeletal Science, University College, London, UK; 3 College of Veterinary Medicine, Michigan State University, East Lansing, MI, 48824, USA; 4 AO Research Institute, AO Foundation, Clavadelerstrasse 8, CH-7270 Davos, Switzerland; 5 Institut für Unfallchirurgische Forschung und Biomechanik, Universitätsklinikum Ulm, Germany; 6 MatSearch, Ch. Jean Pavillard 14, CH-1009 Pully, Switzerland; 7 Institut für Öffentliches Recht, Universität Bonn, Germany; 8 European Academy for the Study of Scientific and Technological Advance, Bad Neuenahr-Ahrweiler, Germany; 9 Ethik im Diskurs, University of Zurich, Zurich, Switzerland; 10 Department of Pharmaceutics and Pharmaceutical Chemistry, University of Utah, Salt Lake City, UT 84112-5820, USA

## Abstract

**Background:**

In an attempt to establish some consensus on the proper use and design of experimental animal models in musculoskeletal research, AOVET (the veterinary specialty group of the AO Foundation) in concert with the AO Research Institute (ARI), and the European Academy for the Study of Scientific and Technological Advance, convened a group of musculoskeletal researchers, veterinarians, legal experts, and ethicists to discuss, in a frank and open forum, the use of animals in musculoskeletal research.

**Methods:**

The group narrowed the field to fracture research. The consensus opinion resulting from this workshop can be summarized as follows:

**Results & Conclusion:**

Anaesthesia and pain management protocols for research animals should follow standard protocols applied in clinical work for the species involved. This will improve morbidity and mortality outcomes. A database should be established to facilitate selection of anaesthesia and pain management protocols for specific experimental surgical procedures and adopted as an International Standard (IS) according to animal species selected. A list of 10 golden rules and requirements for conduction of animal experiments in musculoskeletal research was drawn up comprising 1) Intelligent study designs to receive appropriate answers; 2) Minimal complication rates (5 to max. 10%); 3) Defined end-points for both welfare and scientific outputs analogous to quality assessment (QA) audit of protocols in GLP studies; 4) Sufficient details for materials and methods applied; 5) Potentially confounding variables (genetic background, seasonal, hormonal, size, histological, and biomechanical differences); 6) Post-operative management with emphasis on analgesia and follow-up examinations; 7) Study protocols to satisfy criteria established for a "justified animal study"; 8) Surgical expertise to conduct surgery on animals; 9) Pilot studies as a critical part of model validation and powering of the definitive study design; 10) Criteria for funding agencies to include requirements related to animal experiments as part of the overall scientific proposal review protocols. Such agencies are also encouraged to seriously consider and adopt the recommendations described here when awarding funds for specific projects. Specific new requirements and mandates related both to improving the welfare and scientific rigour of animal-based research models are urgently needed as part of international harmonization of standards.

## Background

As long as improving the human quality of life remains the pre-eminent human ideal, animals will be susceptible to exploitation further the human cause. This simple fact is seen in the evolution of the agrarian civilization, animal domestication, and more recently, in animal use for biomedical research. The remarkable recent developments in human and animal genomics and subsequent explosion in deliberate mutants (e.g., animal knock-outs and knock-ins) will only increase the intensity of animal model development to benefit the human condition. Animal rights and use in this regard depend upon the potential for human benefit and justification for their exploitation. The use of animal models in biomedical research has a long and storied history. Indeed, the first recorded use of animals in research dates to the fourth century B.C. when *Aristotle *studied the anatomical differences among various animal species by dissecting them [1]. Since that time, research using animals has produced significant advances in all areas of medicine [1-6]. Several dedicated compendia describing relevant animal models in biomedical and specific to orthopaedic research are available [7-14]. Significantly, as the limitations and inaccuracies inherent in translating in vitro model results to in vivo equivalence are more widely documented, the use of experimental animal models is likely to increase.

Central to the acceptance of animals as models of human physiology and pathology is the belief that all animals are so closely linked by the bonds of evolutionary and genetic kinship that information gained from one mammalian species is applicable to others [15]. However, when one examines the vast heterogeneity that exists in the animal kingdom, from gross anatomy to molecular levels, such a generalization seems implausible. A more tempered concept was forwarded in 1929 by the distinguished Danish physiologist, *Krogh A*, who wrote, "For a large number of problems there will be some animal of choice, or a few such animals, in which it can be most conveniently studied" [16]. This has become known as the "August Krogh Principle" and has served as a rationale for the use of animal models in biomedical research. The issue then reduces to which biomedical problems should use which animals for appropriate studies, and what criteria should be applied for such selection.

Although the long history of development and recognition of accepted animal models for the study of specific biological phenomena supports the August krogh Principle, baseless or careless application of this principle often leads to fallacious generalizations: 1) extrapolating experimental findings across species is not always valid [17]; 2) Subtle variations in anatomy and physiology, gait (kinematic and kinetic profile), nutrition (e.g., ruminant versus monogastric), age, and even reproductive cycles among various species can greatly impact the response of a specific species to experimental manipulations of the musculoskeletal system. Thus, the researcher is frequently left with the dilemma of which animal model(s) most accurately represent(s) and reproduce(s) the human condition being investigated (if any), and to what extent the results obtained from these models can be correctly and predictably extrapolated to humans. This scenario often expressed in the literature is typified in a recent cartilage repair animal study [18]:

"Although the repair of articular cartilage defects has been studied in many species including rabbits, goats, and sheep, there is no consensus on the most appropriate animal model....none of these species replicate the anatomical, cellular, and biomechanical properties of the human knee. Therefore, we selected the most closely related species, a nonhuman primate (NHP), that may exhibit a healing response most similar to that of humans...."

Such dilemmas are frequently faced in the selection of appropriate animal models for biomedical research. Relevance to human conditions varies widely.

As important as the purely scientific considerations in selecting the appropriate animal model(s) for fracture healing research are the compelling moral, ethical, and legal issues associated with this decision. While humane care and use of all research animals must remain central to any scientific investigation involving living creatures, less agreement exists world-wide as to universal rules, regulations, and oversight that should govern animal use in biomedical research. Lastly, because of their high intrinsic costs, animal experiments receive a large share of available medically derived research funding. From the perspective of "best use of funds" in an era of highly competitive research funding, animal experiments should also be critically evaluated for their direct relevance, validity and experimental value to the desired overall medical research outcome. A simple cost-to-benefit ratio could be determined for animal models in research if metrics for validating appropriate use could be provided.

To achieve some limited consensus on these critical issues, AOVET (the veterinary specialty group within the AO Foundation), the AO Research Institute (ARI), and the European Academy, sought consensus from a group of musculoskeletal researchers, veterinarians, legal experts, and ethicists from candid discussions on the bases for appropriate use of animals in musculoskeletal research. This document represents a consensus opinion from this workshop, seeking to open a more active dialogue amongst researchers with the clear goal to develop and establish internationally accepted guidelines.

### Defining a "justified animal study for fracture healing"

Few in vitro or non-mammalian surrogate experiments accurately or adequately duplicate, or recapitulate the full physiology of the animal model for fracture research purposes. Many research efforts seek new innovative approaches to in vitro modelling of in vivo complexity without clear progress or improved relevance. Use of animal models to study fracture healing under various stimuli and applied therapeutic methods is often an attempt to most expediently duplicate conditions closest to the human patient.

The *"Medical Research Modernization Committee"*, an organisation of researchers who attributes part of the lack of progress in medical research to the use of inappropriate models makes the following statement, which may have some merit: "Animal research advocates claim that animal models can serve as reliable models of human conditions, permitting invasive studies in "controlled" laboratory environments. However this control is largely illusory, because interspecies differences in anatomy and physiology, differences in cause and course between natural human disease and artificially induced non-human pathology, and stress experienced by animals in laboratories invariably alter research results."  This requires closer comparisons of the value and accuracy of animal models in faithfully representing aspects of human conditions. Such comparison should consider all aspects of the specific animal selected, the problem under study, and the entire spectrum of consequences of applying the results to an equivalent human condition.

Several bone and connective tissue human diseases do not naturally occur in animals (e.g., osteoporosis appears to be human-specific [19]) and if they do, such diseases behave quite differently. In most cases, the disease or issue to be investigated must be artificially and, importantly, acutely induced in the normal, healthy animal that serves as the model. Fracture modelling is more intuitive in this regard; however, healing and fixation under compromised conditions (e.g., advanced age, diabetes, osteoporosis, infection) is less intuitive. Hence, reliably producing a pathological or injury condition in an animal that replicates human aspects of disease initiation and propagation in the context of acute or chronic bone fracture healing is difficult. Similarities and analogues are better found for bone fixation or -healing, cartilage or spine degeneration. However, most animals exhibit cell biology, histology, immunology and biochemistry distinct from humans, different healing processes, unique complications, and non-equivalent tissue structures. Additionally, animal behaviour, therapeutic needs and connective tissue healing responses (e.g., inflammation, soft and skeletal tissue healing mechanisms and kinetics) also differ from those of humans. Lastly, usually healthy, normal, robust animals are used to model or study bone fractures, fixation models, disease and complications often clinically observed in unhealthy, aged or healing-compromised humans. These discrepancies with human reality and treatment need to be better identified in descriptions of the models applied where they compromise the validity of the fracture research results.

Few generally accepted large-animal models for most bone and joint studies of orthopaedic devices are designed to replace clinical evaluation in humans. Many effective animal studies show no direct correlation to results in the most significant test – the human – *and this should be continually emphasised*. Finally, humans live much longer than most animals used for research. For fracture healing as a function of age, animal studies are inadequate to assess long-term infection risks and other long-term complications. The criticism is relevant to cortical bone graft research as well, because many of the same parameters "compatibility, durability, and toxicity" are involved . Fracture treatments are conducted in animal models exhibiting different susceptibilities to infection, intrinsic vascular histologies (e.g., the femur and mandible are more highly vascularised than the fibula), biomechanics (e.g., quadrupeds versus bipeds), wounding and healing histologies and responses, and bone histomorphologies. Significantly, bone defects are created in animals often without significant injury to surrounding nerves and blood vessels, and without loss of surrounding soft tissue. A critical element of bone healing in human traumatic bone fractures is the viability of such soft tissue, particularly blood vessels, around the injury site. This aspect, even though it is essential to understand injury and healing phases, is often overlooked in these model comparisons to human problems.

Orthopaedic researchers should begin again to articulate appropriate selection criteria for animal models, clearly focusing on how to most effectively translate basic research elements, e.g., *in vitro *experiments, cadaveric materials, computer models etc., into appropriate animal models that most reliably translate to both human and animal benefit [20]. This should lead to development of universal hypotheses and compelling requirements to test approaches experimentally only in animal models providing the highest scientifically relevant output directly related to addressing the hypotheses and direct translation to human benefit, with minimized animal detriment. So-called "black box" investigations in which several research variables are combined in "design-driven" or empirically assessed animal experiments may serendipitously lead to clinically interesting results, but they cannot provide mechanistic insight or information relevant to biological, physiological and biochemical understanding. If such treatments fail or behave differently between different animal species and humans, developing a revised research approach that attempts a hypothesis-driven experimental pathway is difficult since few scientific hints or guiding details are provided from the initial animal-based experimental designs. The classic scientific method demands hypothesis-driven experimental design wherein proving a null hypothesis (e.g., possibly a negative outcome) provides as much information for rational experimental re-design as a successful experiment. Approaching clinical problems by considering all research possibilities in careful, scientifically validated ways should be essential. This also includes candid presentation of negative results, typically remaining unpublished to date but, for animal models, important for next-generation researchers in a world-wide network, and for full compliance to animal welfare and bioethics.

### Anesthesia and pain management

*Schuppli CA and FraserD *[21], identified three key factors that impede application of the three "R"s (Refine, Reduce, Replace) recommended for animal model use [22]: 1) incomplete understanding of the three "R"s (especially "refinement"), 2) lack of consensus among ethics committee members on the nature and significance of animal pain and suffering, and 3) lack of consensus as to whether ethics committees should indeed follow a policy of minimizing overall harm to experimental animals [23]. "Refinement" is defined as techniques aiming to minimize animal pain, suffering and distress, such as informed use of anaesthesia and post-operative analgesia to minimize post-surgical pain and distress. Competent anaesthetic and analgesic management, appropriate to the scientific objectives of the fracture models, are clearly integral to the aims of refinement in animal research.

#### Anaesthesia protocols

This relatively sophisticated and advanced area of animal welfare management is often under-appreciated. Anaesthesia protocols best suited for each species should be applied, requiring profound knowledge and specialization in this field. Consequently, animal anaesthetic and analgesic protocols should at least be approved and supervised, if not conducted by an experienced and research-qualified veterinary anaesthesiologist or anaesthetist. Laboratory animal models (mice, rats, rabbits) should enrol a thoroughly trained laboratory specialist. As the number of such specialists is presently relatively low, specialty colleges and laboratory experts should attempt to train veterinarians and scientists interested in this field to fill this important void. Given the potential adverse impact of inadequate or inappropriate anaesthetic and analgesic management on animal recovery, fracture healing and animal welfare, future anaesthetic and pain management protocols used in experimental studies should be required to provide full details in the "materials and methods" section of all publications.

#### Pain management

Pre-requisites for adequate pain management in experimental animals are: 1) sound knowledge of pain pathophysiology, 2) sound knowledge of pharmacological/non-pharmacological analgesic strategies, and 3) the ability to identify and evaluate pain in specific species and individual animals.

#### Sound knowledge of pain pathophysiology

Poorly managed pain has many adverse consequences that subsequently impact the validity and success of musculoskeletal research projects, and is known to significantly increase intra- and post- operative morbidity and mortality. Researchers must be familiar with the basic anatomy, physiology, pathophysiology and modulation of nociceptive transmission, including key concepts of peripheral and central sensitisation, multi-modal analgesia, and pre-emptive analgesia. These are summarized in the International Association for the Study of Pain core curriculum for Professional Education in Pain [23].

#### Sound knowledge and application of pre-emptive and multi-modal analgesic strategies

Consistent with multi-modal analgesia, any potentially painful surgical procedure should consider inclusion of all classes of analgesic agents: non-steroidal anti-inflammatory agents (NSAID's), opioids, NMDA antagonists (e.g., ketamine), membrane stabilising agents (local anaesthetics), and α_2 _agonists. Potential direct and indirect effects of each class of analgesic agents on the biological mechanisms under investigation should also be considered. Concerns have been raised about the potential adverse effects of NSAID's on bone healing and metabolism related to signal transduction of pro-inflammatory and related cell signalling cascades.

The pharmacokinetic profile, efficacy and safety of analgesic drugs vary widely between species. Thus, researchers planning animal analgesic programs must be familiar with literature relevant to the experimental species used. Researchers are encouraged to consult sources of information related to rodent and lagomorph anesthesia and pain management for guidelines relating to each species [24,25]. Further research is required to define and improve safe and efficacious analgesic regimes for certain species used in musculoskeletal research (e.g., sheep and rabbits), as the current paucity of pre-clinical and clinical data precludes optimal use of all analgesic strategies currently in use in humans and other animal species.

As analgesic agents are administered to animals by various routes, a combination of systemic and other routes should be considered when planning an analgesic strategy for a particular experimental procedure. Researchers should be familiar with implementing all potential routes of analgesic administration.

#### Ability to identify and evaluate pain in specific species and individual animals

No "gold standard" exists for measuring or assessing animal pain. It is well-recognised that species, gender, age, breed, environment and various stressors influence pain behaviour. It has been generally recognized that the greatest gap in our knowledge of animal pain is the lack of assessment tools.

Peri-operative pain must be assessed frequently by trained, experienced observers who are intimately familiar both with the species, the specific surgical intervention and, ideally, with the individual animals used in the study. The most frequently used subjective scoring systems used for evaluation of animal pain include visual analogue scales, simple descriptive scales, numerical rating scales, and verbal rating or ordinal scales, all of which have inherent limitations. While they can provide useful information when consistently applied by trained, experienced observers intimately familiar with the animals being assessed, their very subjectivity makes them unreliable when used by multiple or poorly experienced observers. Subjective scales used in pain assessment of sheep used in musculoskeletal research have been published [26,27]. Measurement of "objective" physiological variables such as heart rate, respiratory rate, blood pressure, humoral factors including plasma cortisol or endorphin levels, as well as pupil diameter have been shown to be inconsistent and insensitive indicators of pain severity in animals. Systems such as the Glasgow Pain Scale [28], and the University of Melbourne Pain Scale[29] have been developed and thoroughly validated in dogs. No such multidimensional systems have been developed to date for evaluation of pain in experimental laboratory animal species.

Objective pain assessment systems have been developed for certain animal species (dogs, laboratory rats [25,30,31], horses [32], and lambs [33]). Further studies are clearly indicated to define and improve validated, sensitive methods for evaluating pain severity in all research animal species. Procedure-specific, objective, behaviour-based pain assessment systems require development and implementation in species used in musculoskeletal research. Researchers are encouraged to consult information on this subject available on the world-wide web. Further research should critically evaluate all information available and define valid and sensitive methods for evaluating pain severity in all animal species used in research.

### Considerations for establishing standards in surgical, biological and mechanical aspects of in vivo models for research in mechano-regulation of bone repair

Bone, both as a material and structural organ, is acutely responsive to changes in both the biological and mechanical environments. The specific architecture of individual bones is attained as a consequence of a combination of a genetically determined template and prevailing mechanical demands. The base structure of the cortex in dogs (and humans) is composed of secondary osteones, whereas sheep cortex comprises predominantly lamellar bone. However, during bone healing and remodeling, secondary osteones are activated in all these species.

The long-standing law of skeletal structural optimization "Wolff's Law" arose from observations that, as a structure, bone actively adjusts both mass and architecture in relation to the magnitude and direction of applied loads [34]. However, both animals and human individuals have very different base levels of bone mass. Thus, the genotype may influence the specific structural and material features: material properties, for example, may differ by as much as 200% in different laboratory mice strains, related to structural morphology [35]. Furthermore, these differences may be compounded by additional genotype-related responses to mechanical stimuli. This has been demonstrated elegantly in work by *Judex S, et al*. [36]. Continual deformation of bone incurred with loading, as a result of both gravitational forces and muscle action associated with everyday activities, has traditionally been thought to result in micro-damage to the bone matrix. This is seen as micro-cracking. Micro-cracks within bone as a material normally initiate a remodeling process that results in repair through secondary osteonal bone formation [37]. Levels of micro-cracking increase with increasing mechanical demand over a short time course. Increase in mechanical demand over an appropriate time course will initiate structural adaptation. Recent work indicates that this generalization is questionable and some genotypes are "responders" to changes in loading and consequent adaptation, whereas others are not [36].

Gross fracture of bone can occur through either accumulation of fatigue damage or as a consequence of monotonic failure. The repair processes of bone as a structure are modulated through the prevailing mechanical environment. Two basic patterns of repair occur and are dependent upon the level of inter-fragmentary stability. A high level of inter-fragmentary stability as a consequence of rigid fixation induces direct bone repair. This involves osteonal remodeling across the fracture line where fragments are in direct contact and woven bone formation within gaps. Minimal callus formation occurs; the healing process takes place over a long time period. This type of healing is typically achieved with rigid internal fixation devices such as plates. In contrast, where the fragments are stabilized with less rigid fixation, typically external fixation, intramedullary nailing, braces and casts, repair occurs through indirect bone repair, mediated through formation of periosteal and endosteal bridging callus and differentiation of the early fracture hematoma. The rate of repair in terms of the distribution of callus and rate of tissue differentiation is acutely sensitive to the specific mechanical environment at the fracture site. Biological factors, including effects of drugs used for control of inflammation and pain may also modulate the repair process. In intact bone, the pathway for mechano-transduction has been shown to involve the estrogen receptor [38]. Additionally it is known that hormone levels may also modulate bone healing [39]. In research related to mechanisms involved in progression of bone repair, the specific hypotheses may, for example, relate to the biological cascade involved with osteogenesis, or may be more aligned to understanding the mechanics of the fixation device or indeed both. Models that investigate fundamental molecular mechanisms might best use rodent (e.g., murine) species for which the most comprehensive full portfolio of molecular and genetic probes, cell lines, and genetic knock-out/knock-in animals are available. In contrast, for biomechanical studies or those related to biotechnology (e.g., biomaterials) and functional outcomes, a large animal model provides the ability to evaluate devices used in humans more realistically.

#### The role of in vivo models in bone repair research

As indicated above, the entire animal experimental system remains an essential biomedical research component that to date, despite many efforts to find effective surrogate models, is not supplanted in fracture healing research scenarios with non-animal substitutes. Continuous support for the principle of the three R's, veterinary and human clinical caseloads (e.g., spontaneously presenting cases) can be used together as models to advance our understanding and treatment of some fracture healing issues on a comparative basis to benefit both animals and humans. The lack of standardization represents a disadvantage in this strategy. Therefore, induced animal models are also required to test specific hypotheses with scientifically validated criteria for their selection. In addition, methods for data assessment and analysis should aim to minimize confounding variables (see below). The ability to relate data to those from other studies may also be compromised by the use of different models or even the same model used in a different environment. Standardization is therefore critical in maximising the cost benefit of in vivo models.

#### Confounding variables

Fracture research is a relatively small field within all biomedical research, yet the range of models used is perhaps the most diverse. These models vary even within species in terms of the anatomical site used, the husbandry conditions, the genotype (breed or strain), gender, characterization and type of fixation device, and duration of the healing period. The ability to compare data across cohorts, investigators, and species is often facilitated by the use of standard assays and standardized test systems commonly exploited in different laboratories: without common methods and accepted standards, quantitative comparisons are impossible. However, to a large extent, current *in vivo *models used to study bone repair are laboratory-specific, and full description of investigator-recognized features of experimental design and outcomes that can influence the repair processes may not be presented or indeed known. Hence, an important opportunity avails itself to seek consensus on putative international standards that would provide enabling technology for multi-site collaborative studies and direct data comparisons in animal-based bone-healing models.

The age of different species likely impacts the physiological pattern and rate of healing [40], but due to the expense of using geriatric animal models (e.g., inbred research-quality young rabbits cost roughly one-tenth of 2-year old rabbits), little is really known about this important variable well-known in humans to profoundly affect healing. Different anatomical sites may also influence the pattern of repair; this is multi-factorial, partly because of the unique mechano-biology of different skeletal bones and partly as a consequence of biological differences *per se*. For example the metacarpal/metatarsal bones of small ruminants are fused bones (McIII/IV; MtIII/IV) with minimal soft tissue coverage. Tendons and ligaments are found on the dorsal and palmar/plantar aspects of this bone. The tibia presents a bone anatomy providing a subcutaneous surface craniomedially and muscle cover laterocaudally. By contrast, the femur is predominantly covered by muscle on all aspects. Within specific bones healing may also differ at sites, with predominantly cortical healing at diaphyseal sites, and cancellous healing at metaphyseal sites. The extent of cancellous bone also differs between species and is generally limited in relation to the extent seen in humans.

A further consideration involves size-scale physiological disparities between animals and humans [41], and possible effects on bone neogenesis and fracture healing mechanisms. Experimentally studied critical size bone defects [42] demonstrate a species-dependent healing. A 4-mm circular craniomandibular bone defect in mice and 8-mm defect in rats are often both non-healing without therapeutic intervention at 12–24 weeks. However, a 8-mm diameter defect in a larger animal model (e.g., rabbit, dog, sheep) will heal spontaneously since the surrounding soft tissue and remaining perimeter bone interfacial area are better able to supply signals and regenerative milieu to this relatively smaller defect volume. Critical size calvarial defects increase to 17–35 mm diameters in larger animals (guinea pigs, rabbits, dogs, sheep). The linear dimensions of a "critical" defect in bone do not seemingly translate simply across models, nor to humans, nor across different bone defect sites (e.g., cranial to long bone). Additionally, the anatomical site of a critical defect has an effect on its size and therefore possible nutrient transport limitations to heal. For example, an 8 mm-diameter circular defect in rat parietal bones will heal spontaneously, whereas an 8 mm-diameter circular defect in human parietal bone will not. These wound site transport scaling effects also have profound impact on the delivery of exogenous therapeutic and biotechnology components (living cells, growth factors, drugs) to fracture or defect sites. One last consideration here is the problem of the likelihood of surrounding soft tissue prolapse into the bone wound site as a function of increasing size. This might be more profound in the human than in smaller mammalian models, hindering healing. Lastly, a common issue relevant to all animal bone models regards the varying degrees of bone wound repair capability observed phylogenetically, specifically the empirically observed compromised ability of spontaneous bone wound healing in higher order species compared to lower phylogenies [43]. This effect extends beyond mammalian species in larger-sized bone wounds relative to wound size: relatively larger bone volumes will regenerate in rodents and amphibians (e.g., newt) compared to higher mammalian species. This further infers that reservoirs of undifferentiated progenitor cells in rodents are perhaps more potent and responsive and quantitatively superior to humans in bone regeneration.

Surgical approaches and the anatomical positioning of the fixation device, the mechanical characteristics of the device and the activity of the animal can all influence the local mechanical environment at the fracture site. Very small differences in mechanical conditions, especially in the early stages of healing (e.g., restricted housing or mobility), can produce statistically significant differences in both rate and patterns of repair. The method of creating a fracture or osteotomy also varies between models. In the Einhorn model in rodents [44], fractures are induced by blunt trauma, using a controlled weight drop as a non-invasive system, and fixation uses an intramedullary device. In other models [45-48], an open surgical osteotomy or drill hole defect is made. Thus, different levels of soft tissue compromise occur, providing different subsequent effects on bone repair processes [49]. Each animal's hormonal and possibly nutritional status can also influence the bone healing process. Hence, little commonality is observed in the literature to date with regard to fracture models along many different variables, and therefore, little meaningful comparison can be made, even between similar species, if standards are not recognized that unite all of these model features.

As part of both ethical and scientific requirements for improving model value to the orthopaedic surgical community, this wide variety of models could be rationalized and refined to provide the basis for developing standards against which specific interventions could be compared directly.

#### Fracture model standardization

Advances in the field of mechanobiology in bone repair could be enhanced through a common consensus for standard animal models for bone healing, fixation techniques, outcome measures and standard comparison metrics. Major research centres involved in this field of research should move to adopt uniform international standards to refine existing models and adopt common usage decision trees, classifications and rationales. This will lead to an overall reduction in numbers of animals used, and concomitant increase in the research impact per animal sacrificed. Data generated from standardized models should also contribute more rapidly to the development of computer modelling to better predict biological processes and thus eventually replace some *in vivo *models.

Funding agencies are strongly encouraged to insist on access to specialist anaesthetists and veterinary surgeons qualified to help identity and use specific models and animal species for specific purposes. They are also encouraged to seriously consider the positions stated in this paper as a basis for quality, outcome accuracy and excellence when awarding funds for specific bone repair research projects.

A consensus related to *in vivo *fracture models studied for specific purposes is required to establish standards for comparative purposes. It seems appropriate (and traditionally consistent) to adapt a large animal model for device and biomechanical tests, and rodent models for molecular mechanisms and molecular genetics of bone healing and repair. Agreement is needed and should be sought in relation to animal species, breed, gender, age and reproductive state where appropriate, and husbandry regimens. Surgical procedures should be standardized to follow state-of-the-art aseptic techniques where possible using jigs and guides to locate the osteotomy/fracture at a precise position and in relation to anatomical landmarks. Osteotomy/fracture gaps also need to be constant in a standard model, where a standard caliper can be applied. This has not been consistently followed or reported in the literature.

Mechanical influences on bone repair are increasingly studied at a molecular and genetic level as well. Fixation systems should allow stiffness to be both controlled and determined, since even small differences in fixator stiffness influence healing significantly [50]. Some limited control of the mechanical environment in rodent models can be achieved by standardization and fixation system stiffness [45]. *Harrison LJ, et al*. used an external fixation system in a rodent model to standardize fixation stiffness, also allowing a standard plane of section to be taken [51]. This system was used to determine distribution of tissues in the standard section. Such sample analysis also requires careful consideration of methods used, as facture healing results in a heterogeneous distribution of tissue, and therefore standardization of positions of sections and tissue samples should be evaluated and tracked carefully.

Animal physical activity levels should also be considered and quantified where possible, as these also modulate the bone repair process [52]. Hence, therapeutic movement and activity needs in different animals and models should be reconsidered. *In vivo *and postmortem assessments should also require a standard approach, for example the use of jigs and guides for obtaining standard view radiographs, DXA and pQCT scans.

Mechanical testing for fracture stiffness and strength again would be optimized if accepted standardized tests were consistently used. At present, various systems for three-and four- point bending and/or torsion as either destructive or non-destructive tests are used.

Standardized biological assessments may be made from histomorphometry and immunohistochemistry. If so, the plane-of-section in relation to the callus must be constant and specified. The standard sampling system must also apply to tissues used for gene expression determinations and protein evaluations. In each model, assessment time points should be part of the agreed standard.

#### Study design

As part of assuring consistent application of the three "R's" approach, larger experimental studies should only be designed after reviewing results from an appropriate pilot study or as a follow-up to previous studies. Proper factorial analyses and statistical determinations (e.g., standard deviations, power analyses) would allow justification of cohort designs, to produce a greater confidence in study outcome, with the objective of reducing animal numbers needed for research. Additionally, milestones and deliverables must be clearly declared in the research proposal, and animal study designs articulated to reach these milestones. Optimal study design includes maximal appropriate exploitation of research animals while simultaneously maintaining their well-being as a priority. Multiple use of animals or bilateral fracture (osteotomy) management must be avoided for welfare reasons, as well as the negative overall influence of abnormal animal weight bearing during the healing phase, distinct from the true human-relevant clinical situation.

#### Surgical expertise and technique

Just as important as having anaesthetic protocols conducted by experienced veterinary anaesthesiologists or laboratory animal specialists is the expertise of the animal surgeon. Animal surgeries should not be performed by human surgeons not entirely familiar with the detailed anatomy and physiology of the species they use for their experiments. This often leads to post-operative complications resulting in unacceptably high losses of animals that are not declared in publications – these animals lose their lives for nothing and go unnoticed and unreported! Especially in fracture treatment studies using surgical implants in large animals, the known risks of post-operative catastrophic failure must be weighed carefully in the model selection. Complication rates should not exceed 5% and should be fully disclosed in all publications so that future studies can be designed to reduce complications and refine models on an evidence basis.

Two authors *(Auer JA, von Rechenberg B) *too often had to make the unfortunate observation that some human surgeons appear to lack compassion for their research animals, which may indirectly and inadvertently produce increased animal suffering. This leads to the logical demand that future research studies in fracture management be conducted only by surgeons with appropriate surgical and research training in relevant animal models, and capabilities to discern model differences for model selection and follow-through.

### Post-operative animal management and evaluation

#### Personnel

The labour associated with managing experimental animal during a study increases proportionally with the size of the animal used. Therefore, an adequate number of qualified staff with clearly defined responsibilities and management structures is important for effective post-operative management especially in large animal models. These personnel are responsible for adequate record-keeping, administering medication, animal surveillance and maintaining optimal hygiene and animal welfare conditions. Applying Good Laboratory Practice (GLP) guidelines is useful for structuring personnel, data reporting, quality management, and ensuring clear lines of responsibilities and hierarchy.

#### Pre-operative animal acclimatisation

Pre-operative clinical assessment and acclimatisation is an essential component of successful post-operative management. Subject animals should be accustomed to human contact, the housing facility, and any particular post-operative interventions or treatments. One week has been recommended as a minimum acclimatisation period for ensuring that baseline physiological data may be established [53], but longer (e.g., 3–4 weeks) may be more sensible to ensure that the animals are accustomed to their environment, the facility routines and the personnel. Additionally, during this time, the animal should be thoroughly checked for potential health problems or abnormal behaviour, and triaged if necessary. In the case of sheep, goats, pigs and calves, claws should be inspected and evenly trimmed. The animals should be offered a balanced diet during the entire investigative period and initially subjected to effective antiparasitic management.

#### Medications

As mentioned above, carefully selected and administered analgesic medications for postoperative management are indicated. Some researchers feel that in a purpose-built facility, and with appropriate aseptic technique, antibiotics are not necessary, although this may depend on the animal species. The benefit of this approach is that complex interactions between antibiotics and other drugs, with the wound healing process, need not be considered, and problems associated with antibiotic-resistant bacteria are avoided. Other researchers feel that therapeutic doses of broad-spectrum antibiotics are indicated peri-operatively. Their reasoning is that these drugs do not interfere with bone healing, reduce morbidity and aid in reducing animal loss.

#### Post-operative animal housing

In the immediate post-operative period (4–6 hours), animal subjects should be housed in a closely monitored environment protected from adverse climatic conditions and other stressors including people and other research animals. This is best achieved in modern, purpose-built housing for which guidelines have been established in some countries ([54]. Accessed October, 2006). Some additional design components regarding post-operative housing of large research animals (sheep, goats, pigs, calves) used in fracture research include using individual pens fitted with heat lamps and surveillance cameras.

**Figure 1:**
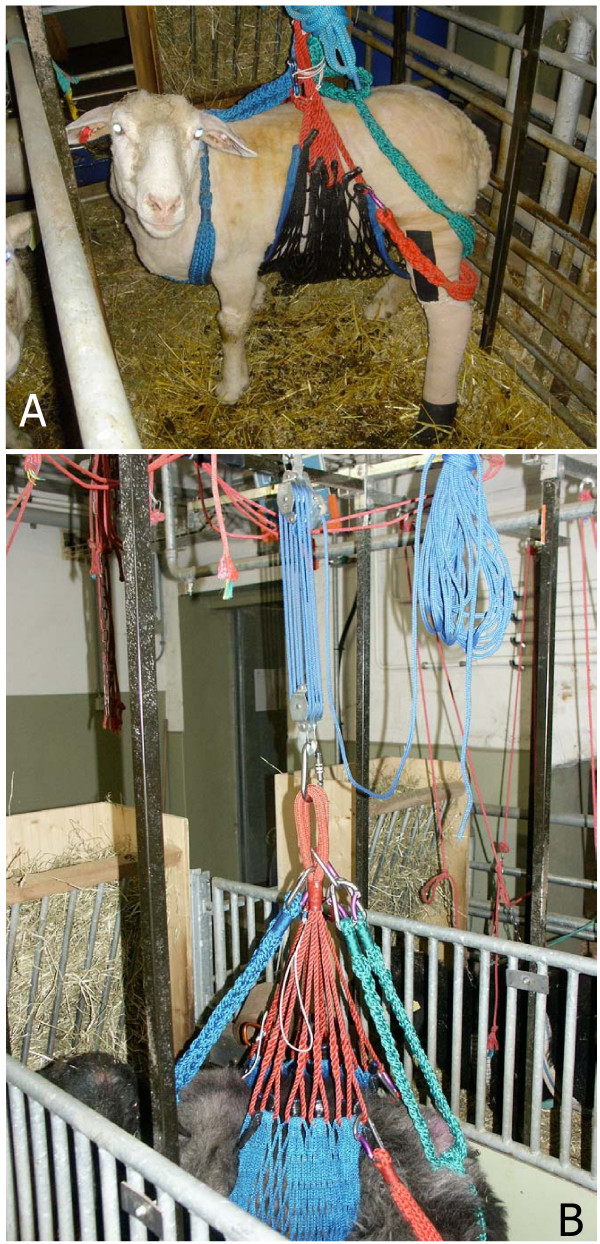
Suspension system for sheep: the net is adapted from horse rescue principles (*Fürst A, et al*.) and was custom made for sheep as a prophylactic measure to avoid refractures of their limbs after (experimental) osteosynthesis. A) The net prevents animals to lie down while still being able to rest and move freely within the stall. The net is loosely wrapped around the rump, the soft belts around the neck and hind limbs of the sheep still allowing them to urinate and defecate without problems. For this reason only female sheep should be utilized for experiments requiring the suspension system. B) The net and belts are bundled above the animal and fixed to a hook on the ceiling with a special device and pulley system that allows free rotation for 360°. A special feeder is used with an extension below where the sheep can rest their heads while sleeping and hanging in the nets. Since social contact is important, animals are never kept alone but always have another sheep as a neighbour.

A contentious issue is the use of supportive slings or harnesses in small ruminants (Fig. 1). These devices, if correctly fitted, do not decrease load-bearing while the animals stand, but they prevent the animal from becoming recumbent or bolting suddenly, and therefore avoid peak torques and loads experienced in limbs. The optimal duration for harness use depends on the type of fracture/defect and repair. The harness or sling should allow movement and rotation of the animal within its stall or box and at least nose and sight contact between individual animals. However, even for the same type of surgery, the optimal duration is unclear. Some researchers favour use only in the immediate postoperative period when poor coordination may be expected. Others prefer to maintain animals in slings until radiographic and clinical evidence indicate that unrestricted activity is not detrimental to the repair. If harnesses are maintained for longer periods, they must be evaluated daily, and changed on regular occasions (e.g., every 3 days) for cleaning and to ensure that rub sores do not develop. Additional application of external coaptation may aid to stabilize the operated limb in the immediate post-operative period. While these harnesses help prevent fixation break down or device failure, restriction of activity and movement also induces a certain type of suffering "accepted" by some species better than others. Nevertheless this aspect must be considered when animals are placed in harnesses.

#### Clinical Assessments

The post-operative assessment itself may stress the animal. Assessment criteria should include food and water intake as well as general attitude, weight bearing and pain score. As mentioned earlier, it is of utmost importance that persons performing post-operative evaluations be familiar with the animal species used and are adequately trained. For example, under Swiss regulations, personnel involved in animal studies are required to attend a training course, and be supervised by a person with demonstrated experience with the animals used. Ideally, post-operative assessments should include ground reaction force measurements in large animals and appropriate activity level measurements for rodents. More invasive evaluations are usually only performed when problems, such as illness develop in the patient.

#### Termination criteria

All animal studies should have clearly defined termination criteria, also called "humane endpoints", at which point animal suffering is no longer justified by the scientific value the animal is providing. Such criteria have been established and are published on the world wide web [55]. They should be communicated and agreed upon by the entire team prior to the start of the study. It is very important that judgement of animal termination be made by a veterinarian not directly vested in the "successful" study completion. A protocol for recovery of any data and collection of tissue samples in the case that an animal needs to be euthanized prematurely should be established to maximize the benefit from unfortunate animal sacrifice. There is a need to establish a consensus on the euthanasia methods used in the different species [56].

### Ethical aspects

Moral issues in using animals for research purposes have long been debated, are controversial, and remain far from solution. We do not intend to re-trace this discussion here, nor do we hope to have developed any new arguments or enlightenment. We take it for granted that using animals as experimental models in biomedical and more specifically in fracture healing research is both in principle morally acceptable, justified by a search for improvements to both animal and human quality of life, and accepted by the general public overall. However, supporting the use of animal models for fracture research does not amount to an excuse for neglecting recognized welfare needs of the animals utilised. Though welfare issues are becoming a customary part of conducting research on animals far more could be done in terms of specifying moral norms and appropriate policies guiding animal research by empirical data. It is our impression that the field of animal welfare science is currently a fast developing area, and that musculoskeletal research can benefit considerably by adopting some of its insights. Therefore, our recommendation to improve anaesthesia and pain management protocols for animals involved in fracture research aims at improving animal welfare irrespective of whether it is influencing research outcomes or not. That such considerations improve the results is an added benefit.

The currently insurmountable plurality of administrative guidelines for animal research that constitute varying animal protection-levels does not, in our view, justify researchers' practice of "regulation-hopping", e.g., to adjust their research efforts to the lowest standard within their reach. Instead, we claim that all researchers should endeavour to actively find solutions for those moral problems generated or intensified through their research. This entails continual engagement in the on-going animal welfare dialogue, and an improved surveillance mechanism to allow more accessibility and visibility to new developments in improving animal welfare. Since this is typically not a formal component of biomedical research education and training, perhaps forms of post-professional or continuing education might be developed to provide such necessary training. The hope that funding bodies and editors may adopt our recommendations for funding- and publishing-decisions is grounded in this view. This would also include journal editors' duties to reject manuscript publications that do not document the application of these ethical standards for the use of experimental animals in studies, and full disclosure of the consequences.

### Need for international harmonization

To date, animal research issues are internationally harmonized on a minimum level. For example, the relevant legal frameworks in Europe – the European Convention for the Protection of Vertebrate Animals and the EC Council Directive 86/609/EEC on the approximation of laws, regulations and administrative provisions of the Member States regarding the protection of animals used for experimental and other scientific purposes -were adopted more than 20 years ago and urgently need modern revision, which is presently under way. Therefore, the Council of Europe as well as the European Community are encouraged to advance harmonized animal welfare standards for scientific research. For this purpose, the legislative competences of the European Community should be expanded to promote effective and binding EC regulation. Obviously, standards on these issues exist on all continents, but at different levels, allowing researchers to export a study to a country with less demanding requirement for animal welfare issues. World-wide harmonization of such issues is desperately needed to stop such behaviour.

As further harmonization on international and super-national levels cannot be achieved in the short run, it is indispensable that, meanwhile, the situation for the proper treatment of experimental animals in fracture research be improved on the respective national levels. To date, most national animal welfare acts are flexible enough to implement the amendments presented in this paper as a kind of good laboratory practice or guidelines clarification while enforcing respective national laws. Thus, national authorizations should in principle only be granted if the intended animal experiment in fracture research observes the improvements as proposed herein. Moreover, ethics committees, wherever involved in the process of authorization, should encourage the respective applicants to address and adhere to the principles proposed in this paper. Where national authorities lack the competence to enforce these principles they should at least be observed by the scientific community on a voluntary basis. Finally, in trans-national research projects, the proposed principles could become a subject of contractual agreement between the scientific partners.

### Future actions

The factors discussed here need to be openly and candidly discussed, possibly amended and eventually agreed upon, and the resulting standardized models for several targeted orthopaedic applications tested widely in a number of laboratories to validate the concept of a reproducible international standard within and between laboratories. This must be followed by a prospective multi-site study to demonstrate the benefits of using such a standard. This should be a funding priority by federal research agencies.

## Authors' contributions

All authors were participants of the invited workshop

JAA: He took the initiative and was the main organizer for the workshop and is the main author of the manuscript.

AG: Contributed to the manuscript

SA: Contributed to the manuscript

SP: Contributed to the manuscript

JP: Contributed to the manuscript

LC: Contributed to the ideas of the workshop

BvR: Contributed to the manuscript

MH: Contributed to the manuscript

ES: Contributed to the manuscript

RMT: Contributed to the ideas of the workshop

FT: Contributed to organization of the workshop and the ideas of the manuscript

KPR: Contributed to the ideas of the workshop

DWG: Contributed to the manuscript and was instrumental in the revision of the manuscript

All authors have read and approved the final manuscript.

## Pre-publication history

The pre-publication history for this paper can be accessed here:


